# Correlation Between Thymus Radiology and Myasthenia Gravis in Clinical Practice

**DOI:** 10.3389/fneur.2018.01173

**Published:** 2019-01-15

**Authors:** Huan Luo, Shanshan Xie, Chao Ma, Wenqiang Zhang, Carsten Tschöpe, Xianen Fa, Jingliang Cheng, Jing Cao

**Affiliations:** ^1^MR Department, The First Affiliated Hospital of Zhengzhou University, Zhengzhou, China; ^2^Department of Ophthalmology, Campus Virchow, Charité - Universitätsmedizin Berlin, Berlin, Germany; ^3^Department of Human Anatomy, School of Basic Medical Sciences, Zhengzhou University, Zhengzhou, China; ^4^Department of Cardiothoracic Surgery, The Second Affiliated Hospital of Zhengzhou University, Zhengzhou, China; ^5^Department of Cardiology, Campus Virchow, Charité - Universitätsmedizin Berlin, Berlin, Germany; ^6^German Centre for Cardiovascular Research (DZHK), Partner Site Berlin, Berlin, Germany

**Keywords:** myasthenia gravis, computed tomography, magnetic resonance imaging, radiology, histology, thymoma

## Abstract

**Background:** The ability to distinguish between a normal thymus, thymic hyperplasia, and thymoma should aid in clinical management and decision making for patients with myasthenia gravis (MG). We sought to determine the accuracy of routine radiological examinations in predicting thymic pathology.

**Methods:** We retrospectively analyzed the records of patients with MG who had undergone thymectomy from the Second Affiliated Hospital of Zhengzhou University. Each patient received at least one initial radiological diagnosis and one histological diagnosis, and the patients were classified into the all-patient, CT, contrast CT, and MRI groups. The sensitivity, accuracy and specificity of each group were calculated for different histological types.

**Results:** This study included 114 patients. All sensitivity, specificity and accuracy values except for sensitivity to hyperplasia in each group for different histological types were satisfactory. MRI had higher sensitivity (68.4, 95% CI: 43.5–87.4%) to histological hyperplasia than did CT (14.3, 95% CI: 0.4–57.9%) and contrast CT (26.7, 95% CI: 7.8–55.1%). Contrast CT had higher specificity (97.9, 95% CI: 88.9–99.95%) for histological hyperplasia than did MRI (88.5, 95% CI: 69.9–97.6%).

**Discussion:** For patients with MG, CT, contrast CT, and MRI examinations can effectively identify thymoma. Additionally, compared with CT or contrast CT, MRI may have a stronger ability to distinguish thymoma and detect hyperplasia.

## Introduction

Myasthenia gravis (MG) is a long-term neuromuscular disease that causes varying degrees of muscle weakness, most commonly affecting the eyes, face, and muscles related to swallowing. MG can cause symptoms including blurred or double vision; ptosis; a change in facial expression; difficulty swallowing; shortness of breath; impaired speech (dysarthria); and weakness in the arms, hands, fingers, legs, and neck (1). MG is regarded as an autoimmune disease because antibodies block or destroy nicotinic receptors in neuromuscular junctions, thereby preventing nerve pulses from stimulating muscle contraction. Most treatments for MG comprise acetylcholinesterase inhibitors, such as mestinon (pyridostigmine), and immunosuppressive drugs, such as prednisone or azathioprine (2). Additionally, a thymectomy may improve symptoms for some patients (2).

A doctor may perform or order several tests to confirm the diagnosis (3, 4). (1) For physical and neurological examinations, a physician first reviews an individual's medical history and conducts a physical examination. In a neurological examination, physicians check for muscle strength and tone, coordination, and the sense of touch and look for impairments in eye movement. (2) An edrophonium test involves injections of edrophonium chloride to briefly relieve weakness in people with MG. Edrophonium chloride blocks the breakdown of acetylcholine and temporarily increases the levels of acetylcholine at neuromuscular junctions. This test is usually used to test for ocular muscle weakness. (3) A blood test for acetylcholine receptor (AChR) antibodies should be performed. (4) Neurophysiological tests are diagnostic tests that include repetitive nerve stimulation electromyography (RNS-EMG) and single fiber electromyography (SF-EMG). SF-EMG is considered the most sensitive test for MG and detects impaired nerve-to-muscle transmission. (5) Diagnostic imaging of the chest using computed tomography (CT) or magnetic resonance imaging (MRI) may reveal the presence of a thymoma. Weakness symptoms are also common in many other diseases; thus, the diagnosis of MG is often missed or delayed for patients who experience mild weakness or for individuals whose weakness is restricted to only a few muscles (5).

The thymus is a gland that controls immune function and may be associated with MG. Located in the chest behind the sternum, this gland is the largest gland in children (6). The thymus gradually grows until puberty and then decreases in size and is replaced by fat. In many patients with confirmed MG, the thymus remains large. The thymus plays a role in MG, and the thymus may provide false indications for the development of immune cells, eventually leading the immune system to attack its own cells and tissues and produce AChR antibodies, thereby impairing neuromuscular transmission. However, the function of the thymus is not fully understood. Some individuals with MG develop thymomas (a tumor originating from the epithelial cells of the thymus that it is not a carcinoma but may show local invasion) (7, 8).

Therefore, assessing thymus histology is very important for MG prognosis. Clinically, we usually perform a thymus tissue biopsy or post-operative histological examination. Although the accuracy of both methods is very high, these methods are invasive and risky (6). With the development of radiological technology, the clarity and accuracy of radiological imaging have increased, making radiology the preferred approach of clinicians (9, 10). Tsutomu Inaoka demonstrated that chemical-shift MRI is helpful for differentiating TLH from thymic neoplasms, especially for equivocal cases upon CT (9). In addition, A.M. Priola et al. concluded that 2-[18F]-fluoro-2-deoxy-d-glucose (FDG)/positron emission tomography–computed tomography (PET-CT) can be used for differentiating thymoma from thymic carcinoma, for assessing response to induction therapy before planning surgical resection, and for detecting pleural seeding and metastatic disease (10). Advanced radiological technology is used in specialized centers to improve accurate detection of thymic pathology in patients with MG (10). However, these tools are not routinely available. Thus, thorough knowledge of imaging techniques and findings for conditions that can occur in MG is essential for defining the correct diagnosis, preventing unnecessary invasive procedures, and assessing proper treatment in a multimodality approach. In this study, we aimed to determine the diagnostic value of conventional radiology with thoracic CT, contrast CT, or MRI to determine thymic pathology.

## Materials and Methods

### Materials

We retrospectively analyzed the records of patients from the Second Affiliated Hospital of Zhengzhou University from the beginning of 2006 to the end of 2016. First, we collected the data of patients with a diagnosis of MG from the hospital information system and medical record files. Then, we examined these records and ensured that each patient had at least one initial radiological diagnosis (CT, contrast CT or MRI; we ensured that each diagnosis was an initial diagnosis because a single patient could receive multiple types of radiological examinations), an initial histological diagnosis, an AChR antibody measurement, documentation of the age of onset, documentation of the sex of the patient, a preoperative Myasthenia Gravis Foundation of America (MGFA) classification, and documentation of the age at thymectomy. If any of these data were absent, we excluded the patient file. The Second Affiliated Hospital of Zhengzhou University review board approved the study protocol.

### Grouping

After performing the screening process described above, we selected patients who met the inclusion criteria and then grouped them. We classified all patients into the all-patient group, patients who had an initial CT diagnostic report into the CT group, those who had an initial contrast CT diagnosis into the contrast CT group, and patients who had an initial MRI diagnostic report into the MRI group. Because a single patient could have multiple types of initial radiology, some patients were placed in multiple groups. This approach made our study more representative of the typical clinical situation.

### Histological Diagnosis

In the abovementioned four groups, we used only the initial histological diagnosis of each patient, which included thymoma, hyperplasia, or normal. When a report confirmed a tumor (including all histological types), the patient was assigned to a diagnosis of thymoma regardless of the stage, and we used the 2015 revised World Health Organization (WHO) classification (11); Masaoka-Koga staging system (12); and the traditional tumor, nodes, and metastasis (TNM) system (12–14) to perform histological grading and staging of the thymoma. The diagnosis of hyperplasia comprised the following histological categories (15): (1) true hyperplasia, defined as an increase in the weight and volume of the histologically normal thymus; and (2) follicular hyperplasia, characterized by an increase in the number of lymphoid follicles with a germinal center. Specimens with an incidental germinal center were classified as normal. In the diagnosis of a normal thymus, we included all reports describing the physiological phase of the thymus, namely, “normal thymus,” “no pathological thymus tissue on histological examination” or “atrophic thymus,” because after puberty, the thymus breaks down at different fat replacement rates, making it difficult to distinguish between a normal and atrophic thymus (16). In this study, pathologists were blinded to the radiological findings.

### Radiological Diagnosis

In all groups, when the conclusion of the radiology report was thymoma, the patient was assigned to a diagnosis of thymoma. The diagnosis of hyperplasia included all thymus tissues with “thymic enlargement/thymus enlargement,” “thymic hyperplasia/thymus hyperplasia,” or an increased anterior mediastinum that did not suggest thymoma. The diagnosis of a normal thymus included all reports of “atrophic thymus,” “normal thymus,” “no thymus tissue seen,” and “no mediastinal abnormalities seen.” In the all-patient group, each patient represented one particular method. If multiple initial radiological results were different for a single patient, we selected the diagnosis based on the order of diagnosis: thymoma>hyperplasia>normal. Additionally, in the CT group, we chose only the CT diagnosis. In the contrast CT group, we chose the contrast CT diagnosis. In the MRI group, we chose the MRI diagnosis. Radiologists were blinded to the other radiological and histological results.

### Surgical Technique Used for Thymectomy

For the cases included in this study, we performed all thymectomies using the video-assisted thoracoscopic surgery (VATS) technique (17). This minimally invasive technique involves several tiny incisions in the chest. A camera is inserted through one of the incisions, and surgery is performed with video guidance. The surgeon removes the thymus by using special surgical tools inserted into the other incisions. The goal is to provide the same result as the more invasive transsternal approach with less post-operative discomfort and a quicker recovery (17).

### Statistical Analysis

To analyze agreement between radiological and histological assignments in each group, we assessed the sensitivity, specificity, and accuracy for each diagnosis. Sensitivity and specificity were calculated as a ratio of correct radiological assignments (true positive or true negative) to the number of respective pathological diagnoses. Accuracy was calculated as the proportion of true positive and true negative assignments in all evaluated cases. All statistical analyses of sensitivity, specificity and accuracy were conducted using R language with the epi.tests package. If the sensitivity, specificity, or accuracy of one of two diagnostic methods was not within the confidence interval (CI) of the sensitivity, specificity, or accuracy of the other, then the difference between the two methods was considered statistically significant. The chi-square test was used to compare the ages of the different groups. The distribution of AChR antibody levels between patients with different histological types was determined by Fisher's exact test. *P* < 0.05 was considered statistically significant.

## Results

### Patient Data Inclusion

**Figure 1:**
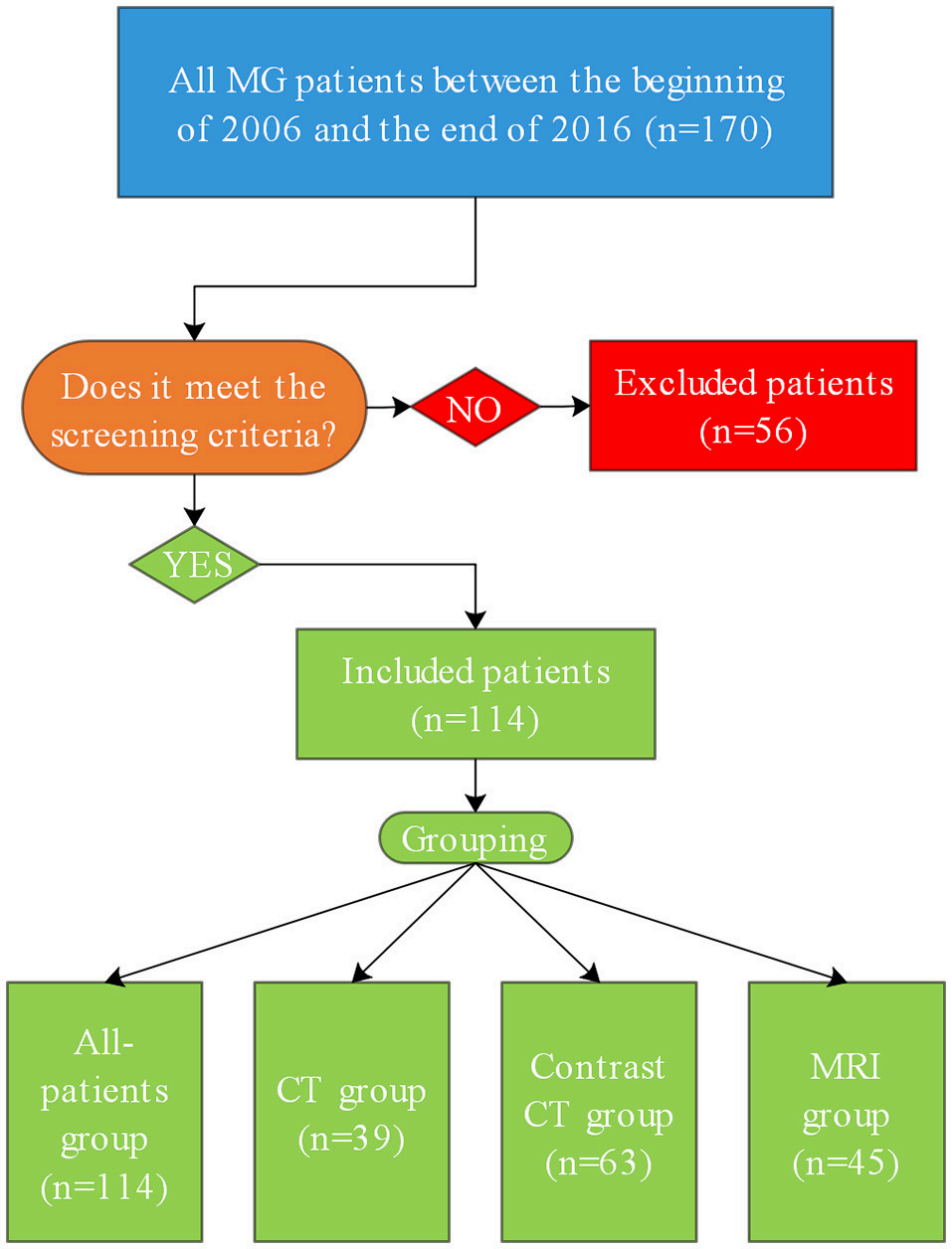
Flowchart showing the inclusion of patients in the study. MG, myasthenia gravis.

We collected data for 170 patients with a diagnosis of MG who were discharged from our hospital from the beginning of 2006 to the end of 2016 in the hospital information system and medical record files. Two patients lacked histological data, 2 patients lacked all initial radiological findings, 31 patients lacked AChR antibody test results, 40 patients lacked age of onset data, 44 patients lacked MGFA classification data, and 4 patients lacked surgery age information. According to the screening criteria, we ultimately removed 56 (32.9%) patients and included 114 (67.1%) patients (Figure 1).

### Histological Grading and Staging of Thymoma

In our study, 25 patients had thymic histology types that were identified as thymoma. According to the newest WHO classification, 2 (8%), 3 (12%), 5 (20%), 13 (52%), and 2 (8%) patients were classified as type A, type AB, type B1, type B2, and type B3, respectively. According to the Masaoka-Koga clinical staging of thymoma, 10 (40%), 11 (44%), 3 (12%), and 1 (4%) individuals were identified as stage I, stage IIA, stage IIB, and stage III, respectively. According to the traditional TNM classification, the majority of cases, namely, 23 (92%), had stage 1 disease; there was 1 (4%) case each of stage 2 and stage 3a disease. Additionally, there were no cases of higher stage disease.

### Grouping

We classified all 114 patients into the all-patient group, which contained 39 (34.2%) patients with a CT result, 63 (55.3%) patients with a contrast CT diagnosis, and 45 (39.5%) patients who had undergone MRI examinations. In this group, 84 (73.7%) patients had only one initial radiological report. Among these patients, there were 13 (11.4%) CT reports, 35 (30.7%) contrast CT reports, and 36 (31.6%) MRI reports. Additionally, 27 (23.7%) patients in this group had two initial radiological reports, and 3 (2.6%) patients underwent all three initial radiological examinations (Table 1).

**Table 1:** Patient characteristics according to the groups.

**Variables**	**All-patient group, *n* = 114**	**CT group, *n* = 39**	**Contrast CT group, *n* = 63**	**MRI group, *n* = 45**
Age of onset, mean (range)	38.8 (1-85)	45.2 (1-75)	44.1 (1-85)	27.4 (1-66)
Age at thymectomy, mean (range)	40.0 (4-86)	47.0 (4-77)	45.0 (4-86)	30.0 (4-68)
**Sex, *n* (%)**				
Men	38 (33.3)	15 (38.5)	21 (33.3)	16 (35.6)
Women	76 (66.7)	24 (61.5)	42 (66.7)	29 (64.4)
**AChR antibody, *n* (%)**				
Positive	96 (84.2)	34 (87.2)	57 (90.5)	34 (75.6)
Negative	18 (15.8)	5 (12.8)	6 (9.5)	11 (24.4)
**Preoperative MGFA classification, *n* (%)**				
Class I	28 (24.6)	10 (25.6)	16 (25.4)	13 (28.9)
Class II a	23 (20.2)	6 (15.4)	11 (17.5)	10 (22.2)
Class II b	51 (44.7)	18 (46.2)	29 (46.0)	18 (40.0)
Class III a	3 (2.6)	1 (2.6)	2 (3.2)	0(0)
Class III b	6 (5.3)	4 (10.3)	2 (3.2)	2 (4.4)
Class IV a	0(0)	0(0)	0(0)	0(0)
Class IV b	0(0)	0(0)	0(0)	0(0)
Class V	3 (2.6)	0(0)	3 (4.8)	2 (4.4)
**Histology, *n* (%)**				
Normal	55 (48.2)	24 (61.5)	29 (46.0)	23 (51.1)
Hyperplasia	34 (29.8)	7 (17.9)	15 (23.8)	19 (42.2)
Thymoma	25 (21.9)	8 (20.5)	19 (30.2)	3 (6.7)
**Radiological sensitivity (95% CI)**				
Normal	90.9% (80.1–97.0%)	91.7% (73.0–99.0%)	93.1% (77.2–99.2%)	87.0% (66.4–97.2%)
Hyperplasia	44.1% (27.2–62.1%)	14.3% (0.4–57.9%)	26.7% (7.8–55.1%)	68.4% (43.5–87.4%)
Thymoma	100.0% (86.3–100.0%)	100.0% (63.1–100.0%)	100.0% (82.4–100.0%)	100.0% (29.2–100.0%)
**Radiological specificity (95% CI)**				
Normal	78.0% (65.3–87.7%)	73.3% (44.9–92.2%)	79.4% (62.1–91.3%)	77.3% (54.6–92.2%)
Hyperplasia	95.0% (87.7–98.6%)	96.9% (83.8–99.9%)	97.9% (88.9–99.95%)	88.5% (69.9–97.6%)
Thymoma	92.1% (84.5–96.8%)	90.3% (74.3–98.0%)	88.6% (75.4–96.2%)	97.6% (87.4–99.9%)
**Radiological accuracy (95% CI)**				
Normal	84.2% (76.2–90.4%)	84.6% (69.5–94.1%)	85.7% (74.6–93.3%)	82.2% (68.0–92.0%)
Hyperplasia	79.8% (71.3–86.8%)	82.1% (66.5–92.5%)	81.0% (69.1–89.8%)	80.0% (65.4–90.4%)
Thymoma	93.9% (87.8–97.5%)	92.3% (79.1–98.4%)	92.1% (82.4–97.4%)	97.8% (88.2–99.9%)

The CT group comprised 39 patients, including 24 patients with an initial contrast CT report and 5 with an initial MRI report. We classified 63 patients into the contrast CT group. Among these patients, 24 had an initial CT report and 7 had an initial MRI result. The MRI group included 45 patients. Of these patients, five had been examined by initial CT, and seven had been examined by initial contrast CT.

The age of onset in the MRI group was significantly lower than that in the other groups (MRI group vs. all-patient group, *P* = 0.0017; MRI group vs. CT group, *P* < 0.0001; MRI group vs. contrast CT group, *P* < 0.0001). There were no significant differences in the age of onset between the remaining three groups (CT group vs. all-patient group, *P* = 0.2120; contrast CT group vs. all-patient group, *P* = 0.2282; contrast CT group vs. CT group, *P* = 0.9904). When we focused on the age at thymectomy (Table 1), the age in the MRI group was lower than that in the other three groups (MRI group vs. all-patient group, *P* = 0.0040; MRI group vs. CT group, *P* < 0.0001; MRI group vs. contrast CT group, *P* < 0.0001). Additionally, there were no significant differences in the age at thymectomy between the remaining three groups (CT group vs. all-patient group, *P* = 0.1763; contrast CT group vs. all-patient group, *P* = 0.2611; contrast CT group vs. CT group, *P* = 0.9704). In all groups, we the number of female patients was approximately twice that of male patients, and the sex distribution between the groups was not significantly different (*P* > 0.05).

Among all patients with MG (*n* = 114), 84.2% had a positive AChR antibody diagnosis (Table 1). In addition, we analyzed the difference in AChR antibody levels between patients with histologically confirmed thymoma and those without histologically confirmed thymoma. The AChR antibody positivity rates were as follows: 96.0% (24/25) for patients with histologically confirmed thymoma (*n* = 25), 88.24% (30/34) for patients with histologically confirmed hyperplasia (*n* = 34) and 76.36% (42/55) for histologically normal patients (*n* = 55). Therefore, the difference in the AChR percentage was non-significant [96.00% (24/25) vs. 88.90% (72/89), *P* = 0.0549] between patients with histologically confirmed thymoma (*n* = 25) and those without histologically confirmed thymoma (*n* = 89).

In our study, approximately 44.7% (51/114) of patients with MG had a preoperative MGFA classification of IIb. The IVa and IVb classifications accounted for the lowest proportions of patients with MG at 0 each, followed by IIIa and V, which were both 2.6% (3/114). The rate of IIIb disease was 5.3% (6/114), and the proportions of I and IIa were similar at 24.6% (28/114) and 20.2% (23/114), respectively (Table 1).

Of the 114 patients with MG enrolled, 55 (48.2%) had normal histological findings, 34 (29.8%) had histological findings of hyperplasia, and 25 (21.9) had thymoma as the histological type (Table 1).

### Radiology Sensitivity

**Figure 2:**
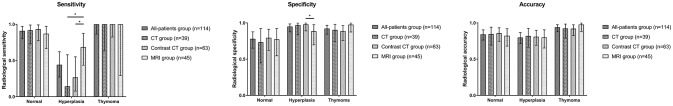
The sensitivity, specificity, and accuracy of each group for the three histological types. Sensitivity: the four methods were highly sensitive to normal histological types, but there was no significant difference. The MRI group had the highest sensitivity (68.4, 95% CI: 43.5–87.4%) in the group of patients with histologically confirmed hyperplasia, and the differences were significant compared with those of the CT group (14.3, 95% CI: 0.4–57.9%) and the contrast CT group (26.7, 95% CI: 7.8–55.1%). There was no significant difference among the CT group, CT group and contrast CT group. All four groups showed 100% sensitivity. For patients with histologically confirmed thymoma, no significant differences were found. Specificity: for hyperplasia patients, the specificity of contrast CT (97.9, 95% CI: 88.9–99.95%) was better than that of MRI (88.5, 95% CI: 69.9–97.6%), and the difference between the two groups was significant. There were no significant differences between the other groups. Accuracy: the four groups showed very similar accuracy measurements between each of the three histological types, and there were no significant differences between the groups.( *Two groups were significantly different).

First, we compared the radiological sensitivity of each group. Regarding histologically normal patients, the contrast CT group had the highest sensitivity (93.1, 95% CI: 77.2–99.2%), followed by the CT group (91.7, 95% CI: 73.0–99.0%), and all-patient group (90.9, 95% CI: 80.1–97.0%). The MRI group had the lowest sensitivity (87.0, 95% CI: 66.4–97.2%). There was no significant difference between the above four groups (Figure 2).

Regarding patients with histologically confirmed hyperplasia, the MRI group had the highest sensitivity (68.4, 95% CI: 43.5–87.4%), which was significantly different from that of the CT group (14.3, 95% CI: 0.4–57.9%), and contrast CT group (26.7, 95% CI: 7.8–55.1%). There was no significant difference between the all-patient, CT, and contrast CT groups (Figure 2).

For patients with histologically confirmed thymoma, the result was simple and clear. All four groups had 100% sensitivity, and there was no significant difference between the groups (Figure 2).

### Radiology Specificity

First, the CT group had the highest specificity in histologically normal patients (79.4, 95% CI: 62.1–91.3%). Second, the lowest specificity was obtained for the CT group (73.3, 95% CI: 44.9–92.2%). The mean specificity of the four groups was not significantly different, and there was no significant difference between the groups (Figure 2).

Regarding patients with hyperplasia, the contrast CT group (97.9, 95% CI: 88.9–99.95%) had the highest specificity, and the MRI group (88.5, 95% CI: 69.9–97.6%) had the lowest specificity. The difference between the two groups was significant (Figure 2).

Regarding patients with histologically confirmed thymoma, the MRI group had the highest specificity, and the contrast CT group had the lowest specificity. No significant difference was evident between the four groups (Figure 2).

### Radiology Accuracy

Interestingly, the four groups had very similar accuracy levels for patients with normal histology, hyperplasia and thymoma, and there was no significant difference between the groups (Figure 2).

## Discussion

To date, due to ethical issues, there are no randomized controlled trials demonstrating that thymectomy provides any benefit to patients with MG. Spillane et al. reported that thymectomy for MG is generally safe and well tolerated and is associated with a sustained improvement in symptoms in the majority of patients (18). However, as many as 20% of patients with generalized MG develop paraneoplastic disease due to thymoma, which represents a clear indication for thymic surgery at any age (8, 19). Another view is that the majority of patients with thymoma should undergo resections. In patients without thymoma, thymectomy is also considered a treatment option. According to this viewpoint, patients with no apparent thymic pathology on radiology can also benefit from thymectomy (20, 21). A histological diagnosis is critical for the MG prognosis. Histological examinations are generally invasive, carry higher risks, and have many contraindications (22). The initial radiological examination of patients with MG is very important for predicting histological outcomes (23). Sussman et al. indicated that thymic radiology is recommended for all patients with MG regardless of antibody status or specific clinical features. The primary value of chest radiology is in identifying patients with thymoma because thymoma has serious management implications (24). Advanced radiological techniques can significantly improve the accuracy of thymic pathology recognition in patients with MG, and their accuracy is almost comparable to that of histological examination; however, radiological pathological testing is performed in specialist centers only, and its use is rarely observed in common clinical practice (10). In this study, we used general clinical radiology to compare histological examinations; calculate the sensitivity, specificity and accuracy of radiological approaches; and evaluate the value of radiological examination for the diagnosis of thymic pathology in patients with MG.

We collected data from 170 patients with MG from medical records. According to the screening criteria, we ultimately included 114 patients. All patient groups represented the natural composition of ordinary patients who visited the hospital. The reason for the establishment of the remaining three groups is straightforward, as indicated by their names.

Notably, we observed a fascinating phenomenon between the groups in terms of the age of onset; the age of onset in the MRI group was significantly younger than that in the other three groups (MRI group vs. all-patient group, *P* = 0.0017; MRI group vs. CT group, *P* < 0.0001; MRI group vs. contrast CT group, *P* < 0.0001). There was no significant difference in the age of onset between the remaining three groups. In other words, the attending physician preferred MRI as the initial radiological test for younger MG patients or younger MG patients preferred MRI as the initial radiology test. We suspect that this association may be related to the following aspects: (1) MRI involves no radiation, and physicians may prefer to use it for young people; (2) older people may have more difficulty in terms of affording the more expensive MRI procedure; and (3) fewer young people have battery-powered devices or iron-metal objects in their bodies. However, no relevant reports or studies were found in the literature. This finding is interesting and can be explored more thoroughly in the future if necessary.

Among the patients with MG included here, female patients accounted for the majority, which was consistent with the literature (25–27).

Among all patients with MG enrolled in this study, three patients with MGFA data were categorized as Class V before surgery; all of these patients underwent contrast CT examinations, 2 underwent MRI examinations, and none underwent CT examinations. These data show that the severity of the condition may be related to the choice of the initial radiological examination by the attending physician. However, the sample size was too small, and a larger sample size is needed to justify this conclusion. In addition, we included no patients with preoperative MGFA data that were categorized as Class IVa and IVb. For this reason, we believe that the sample size may not be large enough or that the preoperative classification was excessively affected by the subjective judgment of the attending physician.

In this study, we also compared patients with thymoma MG with those with non-thymoma MG and found no significant differences in the AChR antibody distribution [patients with thymoma MG vs. patients with non-thymoma MG, 96.00% (24/25) vs. 88.90% (72/89), *P* = 0.0549].

Among the patients with MG with normal histological results, the sensitivity of the three radiological detection methods was higher than 91.7%, and there were no significant differences between the three methods. The specificity of the three radiological examinations was also no <73.3%, and there were no significant differences between the examinations. The accuracy of the three methods was no <82.2%, and there were no significant differences between the three methods.

For patients with a histological diagnosis of hyperplasia, the sensitivity of the three radiological examinations was not high. MRI had the highest sensitivity (68.4, 95% CI: 43.5–87.4%), and significant differences were observed between MRI and CT (14.3, 95% CI: 0.4–57.9%), as well as between MRI and contrast CT (26.7, 95% CI: 7.8–55.1%). However, the specificity of contrast CT (97.9, 95% CI: 88.9–99.95%) for tissue hyperplasia was significantly higher than that of MRI (88.5, 95% CI: 69.9–97.6%) in our study. The three radiological detection methods had an accuracy of no <80.0% for hyperplasia, and there were no significant differences between the three methods. That is, CT and contrast CT had high rates of missed diagnosis of hyperplasia, and the rate of missed diagnosis by MRI was lower than that by CT or contrast CT. However, based on our data, the sensitivity of these three radiological examinations to hyperplasia was still unsatisfactory. The three methods had a low rate of misdiagnosis of hyperplasia, and contrast CT had the lowest rate of missed diagnosis. The diagnostic accuracy of the three methods for hyperplasia was approximately 80.0%, indicating that these methods could distinguish hyperplasia and non-proliferation quite well.

For patients with MG and histological thymoma, all three radiological methods could successfully detect all true positive patients, and specificity was also higher than 88.6%. For diagnostic accuracy, all three methods had good performance. Accuracy was better than 92.1%, with an accuracy of 97.8% for MRI, but differences in the accuracy of the three methods were non-significant.

In the all-patient group, we also observed that regardless of whether the method was sensitive, specific or accurate, it exhibited good performance. This finding indicates that in daily clinical practice, the random selection of initial radiological methods can also obtain good clinical results.

Class I MG is characterized by disturbances that are limited to the oculomotor and levator palpebrae muscles, causing diplopia and ptosis, while the strength of all other muscles is normal (28). MG remains purely ocular in 15% of cases (29), but approximately one-half of patients experience progression within 2 years (30). The myasthenic thymus usually shows histological alterations varying from lymphoid hyperplasia to atrophy or tumor (31), and the production of autoantibodies by thymic B-cells forms the basis of MG pathogenesis (32). The utility of thymectomy in Class I MG is currently debated: some studies support its use (33–35), some oppose it (36, 37), and others are uncertain about its effectiveness (38). Moreover, the relative rarity of this clinical condition prevents the collection of large case series. Our study included 28 patients with Class I MG, with 11 females and 17 males. The average age of onset was 36.2 years old (1–72 years old). Twenty-three patients were positive for AChR antibodies, and 5 were negative. A total of 17 samples were histologically normal, 6 were hyperplastic, and 5 were thymomas. All patients with Class I MG in this study had undergone a thymectomy because although there is no global consensus on the indication of thymectomy in ocular MG, some data support the hypothesis of a beneficial effect in ocular patients, particularly if performed early in the disease course (35). Stable remission was defined as no symptoms or signs of ocular MG upon careful examination for at least 12 months without therapy, except for low-dose steroids (≤10 mg/d methylprednisolone) (35). In our study, 1-year follow-up data were available for 18 of Class I MG patients, 10 of whom achieved complete remission, for a ratio of 55.6% (10/18). The rate might reach 64.3% (9/14) if only patients with histological diagnosis of normal and hyperplastic states were included. Our results are similar to those of previous studies, which confirmed that thymectomy is beneficial for ocular MG (35). Regardless, these data are based on a small sample size, and further verification is needed.

Thymectomy represents the most effective therapeutic option for the treatment of patients with thymoma, assuring a long-term survival in a high percentage of patients, particularly in early stages (39). Regardless, the natural history of thymoma is unpredictable, and relapse may occur in patients initially treated with radical-intent resection; the relapses rate has recently been reported to range from 8 to 30% of patients who undergo this surgery (40–43). The follow-up included clinical evaluation (including neurological evaluation in myasthenia gravis patients) and chest and abdomen CT scan every year (40). Clinical follow-up was performed in this study, 2-year follow-up data were available for 14 of thymoma patients. Among them, 6 cases had recurrence, and the recurrence rate reached 42.9% (6/14), higher than in previous reports. The reason may be that the sample size was too small. These six relapsed patients refused to undergo reoperation but continued medical treatment, and the therapeutic effect needs further observation. According to Marulli et al. reoperation for recurrent thymoma is effective and safe, achieving prolonged survival (40).

The pathogenesis of MG depends upon the target and isotype of the autoantibodies (44). Most cases are caused by immunoglobulin (Ig)G1 and IgG3 antibodies against AChR (44). These autoantibodies generate complement-mediated damage and increase the rate of AChR turnover; both of these mechanisms cause loss of AChR from the post-synaptic membrane (44). The thymus gland is involved in many cases, and experimental and genetic approaches for understanding the failure of immune tolerance to AChR are available (44). Previous studies suggest that AChR autoantibodies are present in approximately 80% of cases of generalized MG but are detected in only 50% of ocular MG patients (26, 27), (45–47). In this study, 18 patients were AChR antibody negative (15.8%), including 5 in Class I, 4 in Class IIA, 8 in Class IIB, and 1 in Class IIIB. There were 8 males and 10 females. The average age was 31 years old (6–61 years old). Moreover, the histology of 13 cases was normal, with 4 being hyperplasia and 1 thymoma. In the 13 patients with normal histology; 3 underwent CT examination that resulted in correct prediction in 2 cases (66.7%, 2/3) and incorrect prediction in 1 (hyperplasia). Four patients underwent contrast CT examination, which successfully predicted 3 cases (75.0%, 3/4) and mispredicted 1 case (hyperplasia). In addition, 9 patients underwent MRI, the finding of which was correct in 8 cases (88.9%, 8/9) and incorrect in 1 case (hyperplasia). In 4 patients with histological hyperplasia, 1 underwent CT scan that wrongly predicted the histological type as thymoma, 1 underwent Contrast CT examination with normal histology reported and 2 underwent MRI that correctly predicted hyperplasia. The only patient with a histological diagnosis of thymoma underwent CT and contrast CT, both of which successfully identified thymoma. Regarding seronegative patient imaging features, although our sample size was not large, the results are still meaningful. However, a larger sample size and in-depth research are needed.

Importantly, most individuals with MG have abnormally elevated levels of AChR antibodies (26). A second antibody—antimuscle specific kinase (MuSK) antibody—is present in approximately half of individuals with MG who do not have AChR antibodies (26). A blood test can also detect this antibody. However, in some individuals with MG, neither of these antibodies are present (26). These individuals have double (AChR/MuSK) seronegative (negative antibody) MG (26). In our study, not every patient had MuSK antibody data; thus, we excluded these data from the study.

In conclusion, our study provides evidence that all three radiological examinations can effectively identify thymoma. Additionally, MRI is 100% sensitive to thymoma, and MRI is better than the other methods in terms of thymoma specificity and accuracy. Therefore, MRI may have a stronger ability to distinguish thymoma. For hyperplasia, MRI has higher sensitivity than does CT and contrast CT, and the difference is obvious. Although MRI appears to exhibit slightly lower specificity than does contrast CT, this gap is very small compared to the advantage that MRI has over contrast CT in terms of sensitivity. Thus, performing MRI on patients with MG may be better for identifying hyperplasia. According to our data, we recommend MRI as an initial imaging method after a patient with MG is admitted to the hospital. However, our study has some limitations. This research was a retrospective study of a series of highly selected patients with MG. More than 30% of patients were excluded, and insufficient patient MuSK antibody data may have affected our results. Moreover, imaging staging and follow-up results were insufficient. Histological reports were provided by different experts, and the preoperative MGFA diagnosis was highly subjective. In addition, more young people in our study had undergone MRI, which may have had an unknown effect on the outcome. We wish to avoid the abovementioned limitations. Nevertheless, these limitations may be desirable because the goal of this research is to analyze the information available in everyday clinical practice.

## Author Contributions

HL, SX, and CM organized and wrote the manuscript. WZ collected the clinical data. HL designed and produced the figures. WZ and JCh contributed to the literature research for the manuscript. CT, XF, and JCa edited the manuscript for grammar. SX revised the manuscript. All authors reviewed the manuscript and approved the manuscript for publication.

### Conflict of Interest Statement

The authors declare that the research was conducted in the absence of any commercial or financial relationships that could be construed as a potential conflict of interest.
